# Levels of Serum 25(OH)VD_3_, HIF-1*α*, VEGF, vWf, and IGF-1 and Their Correlation in Type 2 Diabetes Patients with Different Urine Albumin Creatinine Ratio

**DOI:** 10.1155/2016/1925424

**Published:** 2016-03-16

**Authors:** Ying Shao, Chuan Lv, Qin Yuan, Qiuyue Wang

**Affiliations:** Department of Endocrinology, First Affiliated Hospital of China Medical University, Shenyang, Liaoning 110001, China

## Abstract

*Objective.* To investigate changes in serum 25(OH)VD_3_, HIF-1*α*, VEGF, vWf, IGF-1, and their correlation in type 2 diabetes patients at different stages of diabetic kidney disease (DKD).* Methods.* 502 type 2 diabetes patients were divided into three groups: Normoalbuminuric group (201 patients), Microalbuminuric group (171 patients), and Macroalbuminuric group (130 patients). Serum 25-hydroxyvitamin D_3_ [25(OH)VD_3_] was measured by chemiluminescence. Serum hypoxia-inducible factor-1*α* (HIF-1*α*), vascular endothelial growth factor (VEGF), von Willebrand factor (vWf), and insulin-like growth factor-1 (IGF-1) were determined by enzyme-linked immunosorbent assay. We detected the aforementioned serum factors in all cases and 224 control subjects.* Results.* Serum HIF-1*α*, VEGF, vWf, and IGF-1 in type 2 diabetes patients were significantly higher than those in the control group and increased with the increase of Ln(ACR), respectively (*P* < 0.001). Serum 25(OH)VD_3_ was significantly lower in type 2 diabetes patients and decreased with the increase of Ln(ACR) (*P* < 0.001). Ln(ACR) was positively correlated with duration, HbA1c, Scr, BUN, TC, LDL, TG, UA, HIF-1*α*, VEGF, IGF-1, vWf, and Fg and negatively correlated with 25(OH)VD_3_ and eGFR.* Conclusion*. Serum HIF-1*α*, VEGF, vWf, and IGF-1 may be involved in DKD process through inflammation, angiogenesis, and endothelial injury. Serum 25(OH)VD_3_ may have protective effects on DKD partly by inhibiting inflammation, abnormal angiogenesis, and vascular endothelial dysfunction.

## 1. Introduction

Diabetic kidney disease (DKD) is one of the most common complications of type 2 diabetes, which is the main cause of end-stage renal disease in developed countries. It is characterized by glomerular hypertrophy, basement membrane thickening, and excessive extracellular matrix deposition and ultimately results in glomerulosclerosis and renal interstitial fibrosis. Previous studies have pointed out that the renin-angiotensin system (RAS) is the major mediator of development and progression of DKD [1]. In recent years, more and more reports support the role of chronic inflammation [1, 2], angiogenesis [3, 4], and vascular endothelial dysfunction [5] in the occurrence and development of DKD.

Hypoxia-inducible factor-1*α* (HIF-1*α*) is a highly oxygen-sensitive monitor of regulatory protein in the body. A large body of evidence indicates that HIF-1*α* is a key inflammatory cytokine of renal sclerosis in hyperglycemic conditions [6]. Vascular endothelial growth factor (VEGF) is the most important angiogenesis factor associated with kidney disease [7]. In the kidney, VEGF is mainly produced in the podocyte, and the expression of VEGF is increased at the early stage of DKD, promoting the formation of new blood vessels [8]. Von Willebrand factor (vWf), released from endothelial cells, is a credible biological marker of endothelial cell injury and dysfunction [9, 10]. Insulin-like growth factor-1 (IGF-1) is a potent mitogen for glomerular mesangial cells which can induce cell migration and stimulate the production of proteoglycan, laminin, fibronectin, and type IV collagen, thereby promoting the development of DKD [11]. Vitamin D is obtained from food and from 7-dehydrocholesterol in the skin. Vitamin D attenuates inflammation via inhibiting prostaglandin synthesis and activation and postpones the progress of DKD by inhibiting the activation of some signaling pathways [12].

Although many DKD mechanisms have been proposed, the definitive pathogenesis of DKD remains to be elucidated. Some researches indicate that IGF-1 stimulates the expression of HIF-1*α* and VEGF through activating the PI3K/Akt/mTOR pathway [13, 14]. Ben-Shoshan et al. [15] have pointed out that 1,25-(OH)_2_D_3_ could inhibit the protein expression of HIF-1*α* by inhibiting transcriptional activity of HIF-1 and its target genes, such as VEGF and endothelin-1 (ET-1), thus delaying the occurrence and development of DKD by reducing angiogenesis and inhibiting cell proliferation. Our study provides relevant information on levels of serum 25(OH)VD_3_, HIF-1*α*, VEGF, vWf, and IGF-1 in a large-scale cohort to further understand the law of their changes and to clarify their correlation in type 2 diabetes patients with different urine albumin creatinine ratio.

## 2. Methods

### 2.1. Subjects

The study was comprised of 502 type 2 diabetes patients (256 males, 246 females) who firstly attended Inpatient Clinic in the Department of Endocrinology of The First Affiliated Hospital of China Medical University from June 2012 to December 2014. Admission standards are as follows: type 2 diabetes was diagnosed based on the American Diabetes Association 2010 criteria. Exclusion standards are as follows: patients with hepatic diseases, other kidney diseases, hypertension, cardiac diseases, rheumatic diseases, neoplastic diseases, and infectious or other endocrine diseases (except diabetes). Patients who used thiazolidinediones or statins were excluded. Also, patients who used agents that could affect glucose metabolism (except antidiabetes agents) such as glucocorticoid or used agents that could affect urinary albumin excretion rate (ACR) such as ACE inhibitor or angiotensin receptor blocker were excluded. Patients were classified into three groups according to ACR: Normoalbuminuric group (ACR < 30 mg/g, 201 patients), Microalbuminuric group (ACR 30–300 mg/g, 171 patients), and Macroalbuminuric group (ACR > 300 mg/g, 130 patients). Subjects in Normoalbuminuric and Microalbuminuric groups were all newly diagnosed diabetes and diabetic kidney disease patients, respectively. There were many diabetic patients with macroalbuminuria (selected in macroalbuminuria group) who had not started treatment with standardized treatment regimens before coming to our hospital. The patients were selected on a consecutive basis. We did not exclude any eligible patients. According to the admission and exclusion standards, 502 subjects were selected and 496 type 2 diabetes patients were excluded. Additionally, 224 age- and sex-matched healthy volunteers (110 males, 114 females) who took part in the physical examination in our hospital were recruited as a control group. All healthy subjects were selected based on the results of careful history and clinical examinations. In all eligible healthy subjects, we randomly selected 224 people. This study was approved by the Ethics Institutional Review Board of China Medical University. Written consent was obtained from all participants.

### 2.2. Measurements

In the department of endocrinology, blood samples were obtained from patients in the morning after 12 h of fasting. Medical history and anthropometric measurements were also recorded on the same day. Serum samples were stored at −80°C until final analyses were carried out. The serum factors were measured by commercial sandwich ELISA kits: HIF-1*α* (product number: KA1247, Abnova, Taiwan, China); VEGF (product number: CSB-E11718h, Cusabio, Wuhan, China); vWf (product number: SU7042, YI HAN BIOLOGY, Shanghai, China); IGF-1 (product number: CSB-E04580h, Cusabio, Wuhan, China). The levels of triglyceride (TG), total cholesterol (TC), low-density lipoprotein (LDL), high-density lipoprotein (HDL), fasting blood glucose (FBG), fasting insulin (FINS), uric acid (UA), serum creatinine (Scr), blood urea nitrogen (BUN), fibrinogen (Fg), and 25-hydroxy vitamin D_3_ [25(OH)VD_3_] were detected in the clinical laboratory of The First Affiliated Hospital of China Medical University. Urinary creatinine was measured by automatic picric colorimetry (Beckman, USA). Glycated hemoglobin (HbA1c) level was measured by an automatic glycosylated hemoglobin analyzer (Bio-Rad, USA). Urinary microalbumin was measured by immune turbidimetry (Beckman Coulter, USA) and urine albumin/creatinine ratio (ACR) was calculated; insulin resistance index (HOMA-IR) = FBG × FINS/22.5. Serum creatinine values were used to calculate eGFR with the Modification of Diet in Renal Disease (MDRD) [16]. All the values obtained were in the expected range. Other diabetic complications were not observed in our study.

### 2.3. Statistical Analysis

The IBM SPSS statistics (V.17.0, IBM Corp., USA, 2008) was used for data analysis. Results were expressed as the mean ± SD for normally distributed values and median (interquartile range) for nonparametric values. Differences between the groups were analyzed by ANOVA, followed by LSD's test for normally distributed values and by the Kruskal-Wallis test for nonparametric values. Variables were analyzed on natural logarithm, if necessary. Correlation analysis of serum HIF-1*α*, VEGF, vWf, IGF-1, 25(OH)VD_3_, age, BMI, Scr, BUN, eGFR, FBG, HbA1c, duration, TC, LDL, TG, HDL, UA, Fg, Ln(ACR), FINS, and HOMA-IR was analyzed by Pearson's correlation analysis. Relationship between ACR and correlative factors of ACR was analyzed by multivariate stepwise regression analysis and principal component. All *P* values reported were two-tailed, and a *P* value of <0.05 was considered statistically significant, while a *P* value of <0.01 was highly significant.

## 3. Results

### 3.1. Clinical Characteristics of Type 2 Diabetes Patients and Healthy Controls

Baseline characteristics of the study population are shown in Table 1. There were no significant difference in age, gender, BMI, SBP, DBP, and HDL among the four groups. Duration, FBG, HbA1c, LDL, TC, TG, UA, Scr, BUN, Fg, Ln(ACR), FINS, and HOMA-IR in Normoalbuminuric group, Microalbuminuric group, and Macroalbuminuric group were significantly higher than those in the control group. In patients with type 2 diabetes, duration, HbA1c, TC, TG, UA, Scr, BUN, Fg, and Ln(ACR) in Microalbuminuric group and Macroalbuminuric group were significantly higher than those in Normoalbuminuric group, while eGFR was lower than that in Normoalbuminuric group. The levels of FBG, LDL, TC, TG, UA, Scr, BUN, Fg, Ln(ACR), FINS, and HOMA-IR in Macroalbuminuric group were significantly higher than those in Microalbuminuric group, while eGFR was lower than that in Microalbuminuric group.

### 3.2. Differences in Serum HIF-1*α*, VEGF, vWf, IGF-1, and 25(OH)VD_3_ Levels according to Albuminuria

Table 1 also shows that the serum levels of HIF-1*α*, VEGF, vWf, and IGF-1 in patients with type 2 diabetes were significantly higher than those in the control group and increased with the increase of Ln(ACR), respectively (*P* < 0.001). However, compared with the control group, serum 25(OH)VD_3_ was significantly lower and decreased with the increase of Ln(ACR) in Normoalbuminuric group, Microalbuminuric group, and Macroalbuminuric group, respectively (*P* < 0.001).

### 3.3. Relationship between Ln(ACR) and Various Factors

Table 2 shows that Ln(ACR) was positively correlated with duration, HbA1c, Scr, BUN, TC, LDL, TG, UA, HIF-1*α*, VEGF, IGF-1, Fg, and vWf and negatively correlated with serum 25(OH)VD_3_ and eGFR (*P* < 0.01).

In Table 3 multiple stepwise regression and principal component regression show that HIF-1*α*, VEGF, vWf, IGF-1, UA, BUN, duration, eGFR, TG, 25(OH)VD_3_, and LDL are the main influencing factors of Ln(ACR). *Y*_Ln(ACR)_ = 1.914 + 1.038_principal  component_ + 0.004_UA_ + 0.207_BUN_ + 0.027_duration_ − 0.008_eGFR_ + 0.114_TG_ − 0.017_25(OH)VD_3__ + 0.113_LDL_ (principal component = 0.803 *∗* HIF-1*α* + 0.864 *∗* VEGF + 0.929 *∗* IGF-1 + 0.725 *∗* vWf) (*P* < 0.05).

### 3.4. Correlation with Serum Levels of HIF-1*α*, VEGF, vWf, IGF-1, and 25(OH)VD_3_ according to Different Urinary Albumin Excretion Rates and Its Correlation with Related Factors

Tables 4 and 5 show that HIF-1*α*, VEGF, IGF-1, and vWf were positively correlated with Scr (*r* = 0.174, 0.253, 0.207, and 0.266, all *P* < 0.001), BUN (*r* = 0.328, 0.367, 0.364, and 0.426, all *P* < 0.001), and Ln(ACR) (*r* = 0.525, 0.715, 0.630, and 0.748, all *P* < 0.001), while HIF-1*α*, VEGF, IGF-1, and vWf were negatively correlated with eGFR (*r* = −0.194, −0.307, −0.246, and −0.322, all *P* < 0.001). In addition, these four factors were positively correlated with each other. Serum 25(OH)VD_3_ was negatively correlated with Scr (*r* = −0.103, *P* = 0.020), BUN (*r* = −0.153, *P* = 0.001), and Ln(ACR) (*r* = −0.285, *P* < 0.001), while it was positively correlated with eGFR (*r* = 0.101, *P* = 0.024). Additionally, serum 25(OH)VD_3_ was negatively correlated with HIF-1*α*, VEGF, IGF-1, and vWf.

## 4. Discussion

Studies have suggested that chronic inflammation, angiogenesis, and vascular endothelial dysfunction play important roles in the occurrence and development of DKD. Our research was a large-scale cohort designed to investigate the importance of serum 25(OH)VD_3_, HIF-1*α*, VEGF, vWf, and IGF-1 in DKD pathogenesis.

Results showed that, compared with the control group, serum levels of HIF-1*α*, VEGF, and vWf were significantly elevated in patients with type 2 diabetes and increased as urinary protein increased. Correlation analysis showed that serum HIF-1*α*, VEGF, and vWf were positively correlated with Ln(ACR), Scr, and BUN, respectively. Principal component regression analysis also pointed out that these serum factors are the important factors associated with the increase in Ln(ACR). This may suggest that serum HIF-1*α*, VEGF, and vWf were independent factors associated with DKD.

In hyperglycemic conditions, both protein and mRNA of HIF-1*α* increased significantly in mesangial cell [17]. Under normal oxygen conditions, advanced glycation end-products could increase the transcriptional activity of HIF-1*α* by adjusting the mitogen activated protein kinase [18]. Additionally, it was confirmed that HIF-1*α* promotes renal fibrosis through activating angiotensin II in vivo and in vitro experiments [19].

Abnormal angiogenesis causes the formation of immature blood vessels, which leads to renal fibrosis and eventually results in the loss of glomerular function in DKD. Both the increased VEGF expression and the decreased endothelial nitric oxide synthase expression promote abnormal blood vessel formation in DKD [3]. Expression of VEGF increases the activation of the PI3K/AKT signaling pathway, resulting in phosphorylation of endothelial nitric oxide synthase and angiogenesis, which promotes the process of DKD.

Serum vWf level is closely correlated with endothelial cell injury. Verrotti et al. [20] have confirmed that endothelial dysfunction and the occurrence of microalbuminuria are closely related in DKD. Endothelial injury is one of the characteristics of DKD, even in the subclinical stage of the disease. High glucose produces peroxides and activates endothelial cell nitric oxide synthase, protein kinase C (PKC), and nuclear factor-*κ*B (NF-*κ*B). This leads to excessive reactive oxygen species and reduces the synthesis of nitric oxide. Transcription factors, such as the NF-*κ*B, induce the expression of inflammatory genes in order to increase the expression of cytokines and adhesion molecules [21]. Cruz et al. [22] believed that elevated blood glucose causes inflammation through a variety of signaling pathways, damaging endothelial cells. This, in turn, increases serum vWf and forms a high coagulation state [23], thus promoting the development of DKD.

Previous studies have pointed out that IGF-1 may play a role in these serum factors in other diseases [24]. Our study found that there were positive correlations between serum IGF-1, HIF-1*α*, VEGF, and vWf, so we speculated that serum IGF-1 may be involved in the occurrence and development of DKD through inflammation, abnormal angiogenesis, and vascular endothelial dysfunction.

Activation of the growth hormone GH/IGF-1 axis has a direct relationship with renal hypertension, proteinuria, and glomerular sclerosis, all of which play key roles in early lesions of DKD. IGF-1 causes renal hypertrophy and alters renal hemodynamics through overexpressing VEGF, promoting oxidative stress and high coagulation state. Elevated levels of IGF-1 in DKD are associated with elevated levels of collagen type IV (collagen IV) and laminin which jointly promote the occurrence and development of DKD [25]. Catrina et al. [26] also confirmed that IGF-1 increases the level of HIF-1 by improving the alpha subunit at the level of translation.

Previous studies have pointed out that vitamin D may play a protective role in DKD. Our study found that serum 25(OH)VD_3_ was negatively correlated with HIF-1*α*, VEGF, and vWf, which indicates that vitamin D reduces proteinuria and delays the progression of DKD maybe partly through inhibiting inflammation, abnormal angiogenesis, and vascular endothelial dysfunction.

Plum and Zella [27] speculated that a lack of vitamin D may be an independent risk factor for proteinuria. After adequate vitamin D treatment, urine protein decreased significantly, and fibrosis cytokines, such as TGF-*β*1 and Smad3, were also significantly decreased, suggesting that treatment with vitamin D could significantly delay the process of DKD. Mao et al. [12] confirmed that serum vitamin D levels were negatively related to inflammatory cytokines in the urine, such as TNF-*α*, interleukin-6 (IL-6), and intercellular adhesion molecule-1 (ICAM-1). Ren et al. [28] suggested that 1,25-(OH)_2_D_3_ may exert its therapeutic effect in diabetic retinopathy by inhibiting the VEGF/TGF-*β* pathway. Similarly, Yildirim et al. [29] also confirmed that 1,25-(OH)_2_D_3_ could downregulate the protein expression of VEGF, thereby reducing angiogenesis and inflammation. Compared to DKD patients with normal vitamin D levels, those with vitamin D deficiency have decreased microvascular endothelial function [30]. Vascular endothelial injury leads to adhesion and activation of inflammatory cytokines, which may increase the expression of vWf and further promote DKD process.

Principal component regression analysis also pointed out that TG, UA, and LDL were independent factors associated with DKD. Sustained high glucose leads to systemic vascular endothelial damage, disorder of lipoprotein, and increased TG level. Abnormal blood lipid also promotes the occurrence and development of DKD [31]. UA promotes DKD via renal tubular interstitial injury [32].

Our study has certain limitations. First, it was a cross-sectional study without follow-up and therefore the significance of the changes of serum HIF-1*α*, VEGF, vWf, IGF-1, and 25(OH)VD_3_ as well as the development of DKD remains unknown. Second, our study observed a correlation between serum HIF-1*α*, VEGF, vWf, IGF-1, and 25(OH)VD_3_ in DKD, but not the modulating mechanism.

In conclusion, increased serum HIF-1*α*, VEGF, vWf, and IGF-1 and decreased serum 25(OH)VD_3_ may have an association with diabetic renal damage in type 2 diabetes patients. Serum HIF-1*α*, VEGF, vWf, and IGF-1 may interact with and promote each other and play important roles in the occurrence and development of DKD. Additionally, the protective effect of vitamin D in DKD may be realized by inhibiting inflammation, abnormal angiogenesis, and vascular endothelial dysfunction.

## Figures and Tables

**Table 1:** Clinical characteristics and relative serum factors concentrations of type 2 diabetes patients and healthy controls.

Variable	Control	Normoalbuminuric	Microalbuminuric	Macroalbuminuric
*N* (M/F)	224 (110/114)	201 (99/102)	171 (88/83)	130 (69/61)
Age (years)	48.32 ± 12.30	49.78 ± 8.02	50.48 ± 11.66	49.54 ± 10.75
Duration (years)	—	7.71 ± 5.00^b^	10.22 ± 6.76^bd^	10.87 ± 5.21^bd^
BMI (Kg/m^2^)	24.39 ± 2.76	24.59 ± 3.03	24.94 ± 3.20	24.84 ± 2.22
FBG (mmol/L)	5.10 ± 0.33	10.12 ± 1.74^b^	11.25 ± 2.88^bd^	10.49 ± 4.15^bf^
SBP (mmHg)	124.66 ± 6.10	125.46 ± 6.15	125.05 ± 6.80	125.80 ± 5.93
DBP (mmHg)	74.94 ± 6.21	75.77 ± 6.03	74.85 ± 6.53	76.01 ± 6.15
HbA1c (%)	5.24 ± 0.27	8.39 ± 1.67^b^	8.75 ± 1.80^bc^	8.93 ± 2.10^bd^
HDL (mmol/L)	1.14 ± 0.20	1.10 ± 0.31	1.13 ± 0.25	1.14 ± 0.27
LDL (mmol/L)	2.54 ± 0.45	3.33 ± 0.58^b^	3.48 ± 0.91^b^	3.67 ± 1.18^bde^
TC (mmol/L)	4.01 ± 0.54	5.00 ± 0.74^b^	5.20 ± 1.10^bc^	5.62 ± 1.06^bdf^
TG (mmol/L)	1.21 ± 0.18	1.73 ± 0.77^b^	2.46 ± 1.48^bd^	2.85 ± 1.85^bdf^
UA (mmol/L)	287.14 ± 27.21	268.37 ± 73.60^b^	301.88 ± 72.42^bd^	333.34 ± 42.44^bdf^
Scr (*μ*mol/L)	56.83 ± 9.05	59.33 ± 9.62^b^	62.69 ± 9.66^bd^	68.29 ± 10.51^bdf^
BUN (mmol/L)	4.74 ± 1.03	5.02 ± 0.86^b^	5.90 ± 0.88^bd^	6.36 ± 1.01^bdf^
eGFR (mL/min/1.73 m^2^)	116.92 (112.86~120.99)	125.10 (120.72~129.49)^b^	114.99 (110.64~119.35)^d^	91.98 (88.53~95.44)^bdf^
Fg (g/L)	2.93 ± 0.77	3.13 ± 0.48^b^	3.48 ± 1.02^bd^	3.67 ± 0.31^bde^
Ln(ACR)	1.72 ± 0.16	2.38 ± 0.45^b^	4.19 ± 0.69^bd^	6.56 ± 0.55^bdf^
FINS (mIU/L)	6.61 (6.47–6.75)	9.46 (8.92–10.00)^b^	10.64 (9.65–11.63)^bc^	9.59 (8.70–10.48)^be^
HOMA-IR	1.53 (1.49–6.75)	4.28 (3.83–4.72)^b^	5.45 (4.82–6.09)^bd^	4.48 (3.95–5.02)^bf^
HIF-1*α* (pg/mL)	16.78 ± 6.73	24.38 ± 7.24^b^	30.35 ± 4.93^bd^	33.22 ± 3.89^bdf^
VEGF (pg/mL)	107.60 ± 28.49	130.55 ± 19.48^b^	144.34 ± 23.70^bd^	181.28 ± 21.97^bdf^
vWf (U/L)	372.62 ± 57.56	481.85 ± 62.94^b^	598.49 ± 55.26^bd^	691.63 ± 64.35^bdf^
IGF-1 (ng/mL)	111.42 ± 49.25	149.39 ± 45.10^b^	194.50 ± 44.34^bd^	236.87 ± 46.22^bdf^
25(OH)VD_3_ (ng/mL)	17.36 ± 6.23	14.86 ± 6.19^b^	12.75 ± 5.74^bd^	10.61 ± 5.04^bdf^

Data are means ± SD for Gaussian variables and median (interquartile range) for non-Gaussian variables.

*Note.* Normoalbuminuric, Microalbuminuric, and Macroalbuminuric versus Control, ^a^ *P* < 0.05, ^b^ *P* < 0.01; Microalbuminuric and Macroalbuminuric versus Normoalbuminuric, ^c^ *P* < 0.05, ^d^ *P* < 0.01; Macroalbuminuric versus Microalbuminuric, ^e^ *P* < 0.05, ^f^ *P* < 0.01.

BMI: body mass index; FBG: fasting blood glucose; SBP: systolic blood pressure; DBP: diastolic blood pressure; HbA1c: glycated hemoglobin; HDL: high-density lipoprotein; LDL: low-density lipoprotein; TC: cholesterol; TG: triglyceride; UA: uric acid; Scr: serum creatinine; BUN: blood urea nitrogen; eGFR: estimated glomerular filtration rate; Fg: fibrinogen; Ln(ACR): Ln Koc of urinary albumin to creatinine ratio; FINS: fasting insulin; HOMA-IR: HOMA insulin resistance index; HIF-1*α*: hypoxia-inducible factor-1*α*; VEGF: vascular endothelial growth factor; vWf: von Willebrand factor; IGF-1: insulin-like growth factor-1; 25(OH)VD_3_: 25-hydroxy vitamin D_3_.

**Table 2:** Relationship between Ln(ACR) and various factors.

Variable	Ln(ACR)	
	*r*	*P*
Duration	0.256	<0.001
HbA1c	0.129	=0.004
Scr	0.326	<0.001
BUN	0.483	<0.001
eGFR	−0.394	<0.001
TC	0.256	<0.001
LDL	0.168	<0.001
TG	0.280	<0.001
UA	0.339	<0.001
HIF-1*α*	0.525	<0.001
VEGF	0.715	<0.001
IGF-1	0.630	<0.001
25(OH)VD_3_	−0.285	<0.001
Fg	0.294	<0.001
vWf	0.748	<0.001

HbA1c: glycated hemoglobin; Scr: serum creatinine; BUN: blood urea nitrogen; eGFR: estimated glomerular filtration rate; TC: cholesterol; LDL: low-density lipoprotein; TG: triglyceride; UA: uric acid; HIF-1*α*: hypoxia-inducible factor-1*α*; VEGF: vascular endothelial growth factor; IGF-1: insulin-like growth factor-1; 25(OH)VD_3_: 25-hydroxy vitamin D_3_; Fg: fibrinogen; vWf: von Willebrand factor.

**Table 3:** Multiple stepwise regression and principal component regression of Ln(ACR) and related factors.

	*B*	Standard error	Standard regression coefficient	*t*	*P*
Constant	1.914	0.429		4.466	<0.001
Principal Component	1.038	0.052	0.592	20.031	<0.001
UA	0.004	0.001	0.164	6.210	<0.001
BUN	0.207	0.045	0.125	4.552	<0.001
Duration	0.027	0.008	0.091	3.572	<0.001
eGFR	−0.008	0.004	−0.139	−5.356	<0.001
TG	0.114	0.032	0.094	3.603	<0.001
25(OH)VD_3_	−0.017	0.007	−0.058	−2.292	=0.022
LDL	0.113	0.050	0.057	2.240	=0.025

Since the values of HIF-1*α*, VEGF, vWF, and IGF-1 were correlated, their unique principal component was substituted for them in the model and the principal component = 0.803 *∗* HIF-1*α* + 0.864 *∗* VEGF + 0.929 *∗* IGF-1 + 0.725 *∗* vWf (HIF-1*α*: hypoxia-inducible factor-1*α*; VEGF: vascular endothelial growth factor; IGF-1: insulin-like growth factor-1; vWf: von Willebrand factor). In the formula, each variable was no longer the original variable but standardized variable and the coefficients before the standardized variables represented the correlation coefficients of principal component and the corresponding original variables. Since only one principal component was extracted among the four serum factors and the correlation coefficients were all close to 1, it showed that the four factors were highly correlated and the component could almost contain all the information of the four variables.

UA: uric acid; BUN: blood urea nitrogen; eGFR: estimated glomerular filtration rate; TG: triglyceride; 25(OH)VD_3_: 25-hydroxy vitamin D_3_; LDL: low-density lipoprotein.

**Table 4:** Correlation with serum levels of HIF-1*α*, VEGF, vWf, IGF-1, and 25(OH)VD_3_.

Variable	HIF-1*α*		VEGF		IGF-1		25(OH)VD_3_		vWf	
	*r*	*P*	*r*	*P*	*r*	*P*	*r*	*P*	*r*	*P*
HIF-1*α*	—	—	0.492	<0.001	0.717	<0.001	−0.228	<0.001	0.463	<0.001
VEGF	0.492	<0.001	—	—	0.823	<0.001	−0.243	<0.001	0.519	<0.001
IGF-1	0.717	<0.001	0.823	<0.001	—	—	−0.238	<0.001	0.504	<0.001
25(OH)VD_3_	−0.228	<0.001	−0.243	<0.001	−0.238	<0.001	—	—	−0.219	<0.001
vWf	0.463	<0.001	0.519	<0.001	0.504	<0.001	−0.219	<0.001	—	—

HIF-1*α*: hypoxia-inducible factor-1*α*; VEGF: vascular endothelial growth factor; IGF-1: insulin-like growth factor-1; 25(OH)VD_3_: 25-hydroxy vitamin D_3_; vWf: von Willebrand factor.

**Table 5:** Relationship between serum levels of HIF-1*α*, VEGF, vWF, IGF-1, and 25(OH)VD_3_ and various factors.

Variable	HIF-1*α*		VEGF		IGF-1		25(OH)VD_3_		vWf	
	*r*	*P*	*r*	*P*	*r*	*P*	*r*	*P*	*r*	*P*
Duration	0.113	=0.011	0.123	=0.006	0.136	=0.002	−0.097	=0.031	0.027	<0.001
FBG	0.098	=0.028	0.024	=0.590	0.103	=0.021	−0.047	=0.298	0.092	=0.039
HbA1c	0.010	=0.824	0.091	=0.040	0.063	=0.156	−0.016	=0.716	0.081	=0.069
HDL	0.090	=0.043	−0.019	=0.672	0.012	=0.794	−0.008	=0.857	0.040	=0.377
LDL	0.111	=0.013	0.151	=0.001	0.131	=0.003	−0.016	=0.726	0.142	=0.001
TC	0.143	=0.001	0.248	<0.001	0.223	<0.001	−0.038	=0.400	0.190	<0.001
TG	0.051	=0.254	0.084	=0.060	0.105	=0.019	−0.084	=0.060	0.293	<0.001
UA	0.038	=0.402	0.201	<0.001	0.180	<0.001	−0.069	=0.123	0.280	<0.001
Scr	0.174	<0.001	0.253	<0.001	0.207	<0.001	−0.103	=0.020	0.266	<0.001
BUN	0.328	<0.001	0.367	<0.001	0.364	<0.001	−0.153	=0.001	0.426	<0.001
eGFR	−0.194	<0.001	−0.307	<0.001	−0.246	<0.001	0.101	0.024	−0.322	<0.001
Fg	0.278	<0.001	0.239	<0.001	0.272	<0.001	−0.067	=0.131	0.248	<0.001
Ln(ACR)	0.525	<0.001	0.715	<0.001	0.630	<0.001	−0.285	<0.001	0.748	<0.001
FINS	0.137	=0.002	−0.087	=0.051	−0.105	=0.019	−0.018	=0.689	0.036	=0.415
HOMA-IR	−0.038	=0.394	−0.058	=0.193	−0.152	=0.001	−0.004	=0.929	0.072	=0.109

FBG: fasting blood glucose; HbA1c: glycated hemoglobin; HDL: high-density lipoprotein; LDL: low-density lipoprotein; TC: cholesterol; TG: triglyceride; UA: uric acid; Scr: serum creatinine; BUN: blood urea nitrogen; eGFR: estimated glomerular filtration rate; Fg: fibrinogen; Ln(ACR): Ln Koc of urinary albumin to creatinine ratio; FINS: fasting insulin; HOMA-IR: HOMA insulin resistance index; HIF-1*α*: hypoxia-inducible factor-1*α*; VEGF: vascular endothelial growth factor; IGF-1: insulin-like growth factor-1; 25(OH)VD_3_: 25-hydroxy vitamin D_3_; vWf: von Willebrand factor.
